# Working memory capacity: the need for process task-analysis

**DOI:** 10.3389/fpsyg.2013.00257

**Published:** 2013-05-09

**Authors:** Marie Arsalidou

**Affiliations:** Diagnostic Imaging, The Hospital for Sick ChildrenToronto, ON, Canada

We use working memory (WM) to follow directions, figure out how much things cost and keep up with a conversation. Keeping up with a conversation at the same time as summing up our groceries cost can be a taunting task for most of us. This is multi-tasking and there is a cognitive cost associated with it. Such cognitive challenge is in part due to limitations of WM capacity. The most widely accepted definition of WM is that it is an ability used to manipulate and hold information in mind for brief periods of time. What we clearly know about WM capacity is that it improves dramatically over childhood and adolescence. Researchers, however, are still engaged in a lively debate aiming to better understand how we should measure WM and what its limits are.

Simmering and Perone (2013) made a noble effort to review data on WM capacity development, and their work will be an important reference for future studies. They reported WM estimates across studies, focusing on verbal and visuo-spatial span tasks, backward span tasks and dual-tasks. Their summary shows that most data were collected from children aged 5 through 12 years. There is so much variability in the findings that understandably, Simmering and Perone (2013) avoided the discussion of WM capacity limits across development. However, if we accept that WM estimates they summarized correspond to the number of items a child can simultaneously hold and manipulate, then average scores can be used to identify the WM capacity limits of each age group (Table 1).

**Table 1:** **Average estimates of WM capacity by Simmering and Perone (2013) and theoretical predictions**.

	**Age/year**							
	**5**	**6**	**7**	**8**	**9**	**10**	**11**	**12**
**TASKS**							
Verbal span tasks	3.78	3.76	3.87	4.17	4.73	4.47	4.81	5.46
Visual-spatial span tasks	2.62	2.64	3.44	4.49	4.35	3.77	6.69	5.58
Backward span tasks	1.70	2.55	3.12	3.70	4.20	3.88	4.93	4.65
Complex or dual-tasks	1.33	2.79	1.73	2.88	3.15	3.14	2.44	3.07
**THEORETICAL PREDICTIONS**							
Pascual-Leone (1970)	2	2	3	3	4	4	5	5
Halford et al. (2007)	3	3	3	3	3	3	4	4

The first quantification of WM capacity limit was given by Miller (1956). He noticed that young adults could simultaneously hold 7 ± 2 units of information and proposed the “magic number seven.” In an influential paper Cowan (2001) reported that the focus of attention lies within active memory and its capacity is limited to four chunks. Recently, in collaboration with a developmental theorist, Cowan claimed that WM and reasoning share related capacity limits across development; WM limits 1, 2, 3, and 4 are reached by 1-, 1.5-, 5-, and 11-year olds, respectively (Halford et al., 2007). There is only one unit change, from 3 to 4, between 5-year olds and 11-year olds (Table 1). Thus, according to the Halford et al. (2007) model children of ages 7–8 and 9–10 years should not exhibit any measurable improvements. Developmental changes are in better agreement with theoretical predictions made by Pascual-Leone over 40 years ago (Table 1). Pascual-Leone (1970) suggests that after the age of three WM capacity grows by one unit every other year until age 15 when it reaches seven units, which is also the limit for adults. In addition to the importance for accounting for age variations, Simmering and Perone (2013) recognized that accounting for cross-task variation is a key limitation in many developmental theories.

Cross-task variations can be accounted for via use of process task-analysis (e.g., Pascual-Leone and Johnson, 1991, 2011). Process task-analysis can be used to create a metric for estimating cognitive load across domains and contexts. It is a rationally based approach used to predict the cognitive demand of a task (e.g., the number of WM capacity units that need to be utilized to solve the task; Pascual-Leone and Johnson, 2005, 2011). It incorporates load information related to situational features, procedural, and figurative sources. Process task-analysis was used in the design of a novel visual-spatial WM capacity paradigm (Arsalidou et al., 2010) and data showed that irrelevant task features improved the assessment of WM capacity across development. Absence of irrelevant features lead to over-performance in younger age groups (Arsalidou et al., 2010). Specifically, using six levels of difficulty (WM demand ranged from 3 to 8) WM scores for 7–8, 9–10, 11–12, 13–14-year olds were 3.25, 4.14, 5.57, and 6.68, respectively (Arsalidou et al., 2010). Identifying WM demand that tasks impose on the individual can improve methodological choices in the design and implementation of tasks with neuroimaging techniques.

Understanding the factors that contribute to task demand can help bridge the gap between real-time behavioral outcomes and brain responses recorded using techniques such as functional magnetic resonance imaging (fMRI). For instance, using the same task (Arsalidou et al., 2010) adults showed comparable behavioral performance for task items with WM demand 3–8, however, activity in the brain showed a significant linear increase in a set of brain areas in prefrontal (middle frontal and cingulate gyri), and posterior (fusiform gyri and inferior parietal lobule) cortices (Arsalidou et al., 2013). Process task-analysis showed the breakdown of procedural and figurative aspects of the task can inform the interpretation of fMRI findings. Particularly, activation patterns showed region-specific roles that give support to two capacity limits for adults; an upper bound of 7 units and a lower bound of 4 units (Arsalidou et al., 2013).

The review by Simmering and Perone (2013) highlights the need for further investigation into WM capacity and the need to understand the underlying processes in order to integrate variability observed in findings. Process task-analysis provides important means with which to specify the components that contribute to cognitive load across tasks, allowing more solid basis for future studies. A universally accepted clear picture of the mechanisms of WM capacity is still ahead of us and there is no doubt that new empirical evidence will fuel the construction of improved theoretical frameworks.
